# bOptimizing atomic force microscopy for characterization of diamond-protein interfaces

**DOI:** 10.1186/1556-276X-6-337

**Published:** 2011-04-14

**Authors:** Bohuslav Rezek, Egor Ukraintsev, Alexander Kromka

**Affiliations:** 1 Institute of Physics, Academy of Sciences of the Czech Republic, Cukrovarnická 10, 16253 Prague 6, Czech Republic

## Abstract

Atomic force microscopy (AFM) in contact mode and tapping mode is employed for high resolution studies of soft organic molecules (fetal bovine serum proteins) on hard inorganic diamond substrates in solution and air. Various effects in morphology and phase measurements related to the cantilever spring constant, amplitude of tip oscillations, surface approach, tip shape and condition are demonstrated and discussed based on the proposed schematic models. We show that both diamond and proteins can be mechanically modified by Si AFM cantilever. We propose how to choose suitable cantilever type, optimize scanning parameters, recognize and minimize various artifacts, and obtain reliable AFM data both in solution and in air to reveal microscopic characteristics of protein-diamond interfaces. We also suggest that monocrystalline diamond is well defined substrate that can be applicable for fundamental studies of molecules on surfaces in general.

## Background

Diamond is an attractive material for merging solid-state and biological systems [1-7]. Polished monocrystalline diamonds (MCDs) exhibit low enough surface roughness (RMS < 1 nm) so that they are suitable for morphology and thickness determination of thin proteins layers [8,9]. Diamond is also chemically inert, yet its surface can be functionalized by different atoms and even organic molecules [1,4] that lead to diverse functionality of diamond surfaces. The most representative and the most widely employed instances are hydrogen and oxygen surface atoms. Hydrogen-terminated diamond (H-diamond) surface is hydrophobic while oxygen-terminated diamond (O-diamond) surface is hydrophilic. Those and other properties of diamond-solution interfaces [10] play an important role when interfaces to biological systems are made. They can be used to induce growth of cellular micro-arrays [5] as well as to fabricate bio-electronic devices [2,3,6,11].

For interfaces employing cells, proteins in the cell medium represent a crucial factor. For instance, presence of fetal bovine serum (FBS) in McCoy's 5A medium (5-15%) promotes selective SAOS-2 cell (sarcoma osteogenic, human osteoblast-like cell line) growth on O-diamond in the case of H-/O-termination micropatterns [5]. This function was understood only after atomic force microscopy (AFM) was applied in solution where it can reveal different protein conformations that are not detectable in air [8,12]. Combination of advanced AFM regimes (including phase imaging and nanoshaving) has shown that the FBS quickly adsorbs on the whole surface, forms 2-4 nm thin layer, yet morphology of the proteins is different on H- and O-diamond [8]. After several days, FBS was shown to form even more complex bi-layer structures [9].

AFM characterizations of soft organic molecules, in particular under such *in situ *conditions, are challenging for many reasons. In general, AFM measurements may generate a number of various artifacts. We denote as artifact a structure or feature in AFM data that is not present in the original material, but generated due to the effect of measurement itself. Most common artifacts are well known and documented [13,14]. They are associated with effects such as hysteresis, piezoelectric creep, thermal drift, cross coupling between various axes [15], nonlinear relationship between cantilever deflection and laser spot movement, or nonlinear detector response [16]. Several tip and scanner artifacts as well as effects of vibrations, feedback, and image processing were also described [17].

Specific types of artifacts are related with AFM on molecules. They may lead to misinterpretation of the dimensions, shape and even presence of molecules. For instance, morphology of organic molecules can be easily influenced by the tip shape that has comparable dimensions to the molecules [18] and by the forces exerted by the tip in both contact mode (CM) and tapping mode (TM) measurements [4,12,19]. The molecules can be even partially or fully removed from the surface using common measurement settings of TM [9]. These effects are particularly pronounced in liquids where Van der Waals forces are more effectively screened and molecule-substrate interaction is thus weaker [12]. Moreover, AFM cantilevers have generally lower quality factor Q when oscillated in solution, hence their sensitivity to forces is decreased.

For those reasons, the AFM measurement must be optimized in a way which minimizes intrusiveness, checks for possible artifacts, and provides a good contrast and spatial resolution in the measured data. The tip-surface interaction can be reduced by adjusting a TM set-point ratio, i.e., ratio of cantilever oscillation amplitude in approached and free state, as close to 1 as possible. However, this may lead to a loss of spatial resolution and true height can anyway only be deduced by extrapolation [4,20]. Novel AFM techniques are constantly being developed to avoid such difficulties, for instance by employing very soft polymers cantilevers [21], low noise all-fiber interferometer as the deflection sensor [22], jumping mode [12], or higher harmonic mode [23]. However, such novel techniques are not widely available and they are often more complicated and hence more difficult to adjust and interpret compared to now common CM and TM regimes.

Here we demonstrate how to resolve different morphology and adhesion of proteins on diamond using regular CM and TM AFM regimes. We use FBS proteins adsorbed on H- and O-diamond as a case study of organic-inorganic systems with general implications for diamond and other inorganic materials employed for bio-interfaces. Such biological interface systems are highly relevant scientifically as well as technologically [24,25]. Characterization and understanding of interactions at organic-inorganic interfaces is extremely important also for human safety [26]. AFM is one the main methods for such studies. We investigate the influence of AFM cantilever and tip properties as well as measurement parameters such as cantilever oscillation amplitude, on the CM and TM AFM characterization of such specimens. General influence of the said parameters is known to an experienced AFM user. However, specific effects that may appear in the measured data are not obvious and not described in the literature. We discuss specific artifacts that may arise, propose schematic models, and provide insights how to optimize AFM for reliable and high resolution measurements of various characteristics on diamond-protein interfaces. We also show advantages of monocrystalline versus nanocrystalline diamond (NCD) substrates.

## Methods

Nominally undoped MCD substrates were chemically cleaned in acids (97.5% H_2_SO_4 _+ 99% powder KNO_3_) at 200°C for 30 min. The surface was then H-terminated in hydrogen microwave plasma at 600°C for 10 min. MCD was photolithographically processed to generate alternating H- and O-terminated patterns of 30 μm widths. A positive photoresist ma-P 1215 (micro resist technology GmbH, Germany) was used. MCD with lithographic mask were treated in oxygen radio-frequency plasma (300 W power, 6 min process time) to selectively oxidize the surface and hence to generate the hydrophilic patterns. Finally the samples were rinsed in stripper solution, de-ionized water and dried. This procedure of selective oxidation of diamond film is well established in the diamond research field and in the literature [5]. For comparison, we have also employed NCD films deposited by microwave plasma chemical vapor deposition on silicon substrates. Deposition procedures of such diamond films are described in detail in Ref. [27].

Proteins were adsorbed on MCD from 15% FBS solution (PAA) in McCoy's 5A medium with stable Glutamine without Phenolred (BioConcept). Typical adsorption time was 10 min. The effect of adsorption time on protein layer structure and thickness was studied earlier [9]. The serum contains several proteins, bovine serum albumin, fibronectin, vitronectin, etc. FBS is heat inactivated (56°C, 25 min) to destroy the immunological components yet preserve the proteins. The samples were fixed inside the fluid cell, which was filled with 1.5 ml FBS solution, and characterized by AFM (Ntegra, NT-MDT). Measurements in solution are important to study real protein conformations. For examples, topography of protein molecules on H-terminated diamond and O-terminated diamond surfaces are different when studied in solution and are similar when studied in air [8]. Some measurements were done also in air; humidity was in the range of 30 ± 15%. All AFM experiments were performed at room temperature.

TM was employed for morphology characterization and CM nanoshaving for thickness determination [4,27]. Three different types of cantilevers were used, ranging from soft (CSG01, NT-MDT, *k *= 0.06 N/m), medium (NSG01, NT-MDT and Multi75Al cantilevers, BudgetSensors, *k *= 3 N/m) to stiff (diamond tip, NaDiaProbes, ADT, *k *= 120 N/m). Cantilever spring constants were not calibrated; they were taken from the manufacturers' specifications.

Free oscillation amplitudes in the range of *A*_0 _= 20-800 nm in air and *A*_0 _= 10-60 nm in solution were used. A feedback gain of FB ~ 0.1-0.5 was typically used to prevent oscillations in feedback loop while providing fast enough height response. If oscillations were observed at FB < 0.1, we repositioned the cantilever in the holder or used new cantilever to avoid loss of spatial resolution at too low FB gain. Measurement set-point ratio was optimized to minimize the influence on the protein layers yet to provide optimal resolution and contrast. The set-point ratio (*r*_SP_) is defined as the ratio between amplitude of oscillation during scanning *A *and free oscillation amplitude *A*_0_. Usually *r*_SP _was in the range of 0.5-0.7. Most of the measurements were performed in solution, where softest tapping correspond to *r*_SP _~ 0.6, measurements with *r*_SP _~ 0.55 may lead to losing of contact within one frame and only *r*_SP _~ 0.5 give stable image for several scans. To have comparable data in air and in solution we used the same free amplitude (50 nm) and *r*_SP _(0.5) for all measurements (except for testing or high amplitude measurements). However, considerable interaction between the tip and surface must be taken into account.

Ntegra NT-MDT software was employed for the data analysis. Tip radius was estimated by a deconvolution analysis. Typical lateral feature size was characterized by Lx values, which were determined using autocorrelation function. Root mean square (RMS) values were employed for characterization of amplitude variations of topography and phase across the surface. RMS values were also used as an error bar of determined layer thickness [8].

## Results

**Figure 1:**
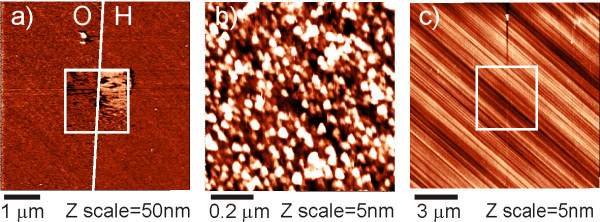
**Influence of cantilever spring constant on FBS layer characterization**. TM AFM images of fetal bovine serum (FBS) on H/O-terminated monocrystalline diamond (MCD) in solution using cantilevers with different spring constants: (a) by CSG01 cantilever, NT-MDT, *k *= 0.06 N/m. Central area was scanned in CM (nanoshaving) across H/O-terminated diamond, the position of the boundary was determined using topography data from a large area 135 × 135 μm^2 ^scan, actual scan size is 10 × 10 μm^2^, *A*_0 _~ 300 nm, energy of cantilever oscillations *E *~ *kA*^2^/2 ~ 10^-15 ^J; (b) on O-diamond by Multi75Al cantilever, Budget Sensors, *k *= 3 N/m, scan size 1 × 1 μm^2^, *A*_0 _~ 50 nm, *E *~ 10^-14 ^J; (c) on O-diamond by NaDiaProbes, ADT, *k *= 120 N/m, scan size 15 × 15 μm^2^, *A*_0 _~ 50 nm, *E *~ 10^-13 ^J, proteins were removed from the central area of 5 × 5 μm^2 ^by CM scan prior to TM.

Figure 1 shows the results of AFM measurements of FBS on MCD in McCoy's solution using cantilevers with different spring constants, from very soft (*k *= 0.06 N/m) to very stiff (*k *= 120 N/m). At first, CM nanoshaving of FBS was performed in the central area of 2 × 2 μm^2 ^on the border between H- and O-terminated surface regions. The position of the boundary was determined using topography data from 135 × 135 μm^2 ^scan. The applied force ranged from 2.5 to 25 nN. The threshold force for protein removal from diamond surface was about 10 nN. This corresponds well to the threshold that has been reported in other experiments [8] and indicates non-covalent adsorption of proteins to the surface [4,28]. The image as shown in Figure 1a was then taken by TM using the low *k *cantilever at the second harmonic resonance frequency (*k *= 0.06 N/m, *f*_2 _~ 30 kHz, *A*_0 _= 300 nm). Amplitude at the first resonance frequency in solution was extremely low (*A*_0 _< 1 nm) even with maximal driving voltage (10 V) and was not usable for measurements. H-terminated MCD surface, where FBS was removed, appears significantly higher (by 10 ± 3 nm) than the original FBS layer. Sometimes steps up to 200 nm in height were observed on H/O- boundary on MCD in solution using CSG01 cantilever at second harmonic resonance frequency. This is artificial as the real height difference between H/O surfaces is <1 nm as obtained using the medium frequency cantilevers both in CM and TM.

Figure 1b shows FBS layer morphology on O-terminated MCD obtained by TM using the medium *k *cantilever (*k *= 3 N/m) at the first resonance frequency (*f *~ 30 kHz) with the amplitude of *A*_0 _~ 50 nm. Using smaller amplitudes resulted in a too noisy image. The morphology is clear with fine features that correspond to previously reported AFM data on FBS [8].

Figure 1c shows TM scan of FBS layer on O-terminated MCD surface using stiff cantilevers (*k *= 120 N/m) at the first resonance frequency (*f *~ 150 kHz) with the amplitude of *A*_0 _~ 50 nm. Again, CM nanoshaving of FBS was performed at first in the central area of 5 × 5 μm^2^. Only MCD surface with ordinary polishing marks without protein molecules is visible. Obviously, the stiff cantilever penetrated and completely removed the FBS layer even in TM.

**Figure 2:**
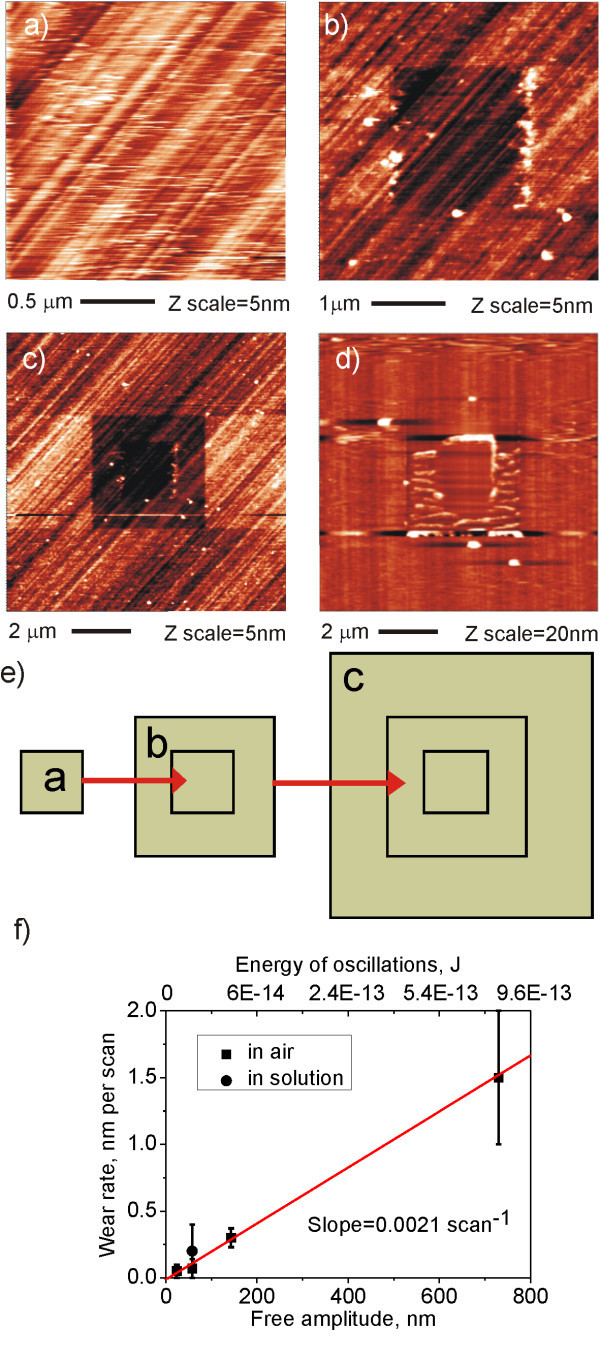
**Wear of protein layers in tapping mode AFM**. AFM image of FBS layer on monocrystalline diamond (MCD) in McCoy medium using NSG01 tip (NT-MDT): (a) The protein layer was removed from 2 × 2 μm^2 ^area in CM (*F *~ 600 nN). (b) The protein layer was partially removed from 4 × 4 μm^2 ^area using 5 scans in TM (*A*_0 _~ 60 nm, *E *~ 10^-15 ^J). (c) The protein layer was measured by TM, scan 10 × 10 μm^2 ^(*A*_0 _~ 60 nm). (d) FBS layer on MCD was studied in CM and TM in air using NSG01 tip. FBS layer was removed after just 2 scans with the highest amplitude (*A*_0 _~ 700 nm, *E *~ 10^-12 ^J). (e) The scheme illustrates the TM nanoshaving experiment as shown in (a-c). (f) Wear rate dependence on free amplitude and oscillation energy for TM nanoshaving of FBS layer adsorbed on MCD using medium *k *(NSG01) cantilevers, *r*_SP _= 0.5.

Figure 2 shows that FBS layer can be removed from MCD in TM in McCoy's solution even when using the medium frequency cantilevers. We applied subsequent scanning as illustrated by the scheme in Figure 2e. First, one 2 × 2 μm^2 ^CM scan with the applied force of *F *~ 600 nN completely removed the FBS layer from the MCD, as shown in Figure 2a. Afterwards, five 4 × 4 μm^2 ^scans in TM with standard amplitude (*A*_0 _~ 60 nm) were made. An image obtained by these scans is shown in Figure 2b. It confirms removal of the FBS layer during the previous CM scan. An overall scan 10 × 10 μm^2 ^with the same amplitude (*A*_0 _~ 60 nm) in Figure 2c reveals that the five previous TM measurements lead to partial removal of FBS film, too. We denote this effect as TM nanoshaving [9]. This effect is more pronounced when stiffer cantilever is used, as demonstrated already in the Figure 1c.

Figure 2d shows results of TM nanoshaving of the FBS layer on MCD using the medium *k *cantilever at high amplitude in air. First, one 2 × 2 μm^2 ^CM scan with the applied force of *F *~ 600 nN completely removed the FBS layer to reveal the diamond substrate. Then, two TM scans with high cantilever amplitude (*A*_0 _~ 700 nm) were performed in the 4 × 4 μm^2 ^area. The final TM AFM image obtained at the standard amplitude (*A*_0 _~ 60 nm) shows that FBS layer was removed also by the medium *k *cantilever at high amplitude in TM. There is some aggregation of FBS noticeable along the scan lines. That is a common effect when non-covalently bound molecules are nanoshaved [27].

The above results can also be used to deduce a wear rate of FBS layers in TM AFM. Figure 2f shows the wear rate of FBS layer on O-diamond using medium *k *cantilevers as a function of oscillation amplitude *A *in solution and in air. Energy of oscillation at specific amplitude is given on the top axis. It is approximated as *E *= *kA*^*2*^*/2 *(simple oscillator equation). More precise estimation requires determining parameters such as quality factor of cantilever and phase of the cantilever relative to the driver [29]. The wear rate ranges between 0.1 and 1.5 nm/scan. The wear rate in solution has higher error bar than in air due to the higher error in the layer thickness determination. The wear rate is linearly proportional to the applied amplitude, in agreement with previous reports [30,31]. The slope of linear regression of the data is 2.1 × 10^-3 ^scan^-1^. It should be noted that this is not wear of individual protein molecules but of the layer adsorbed as a whole from FBS/McCoy's solution that contains diverse organic materials.

**Figure 3:**
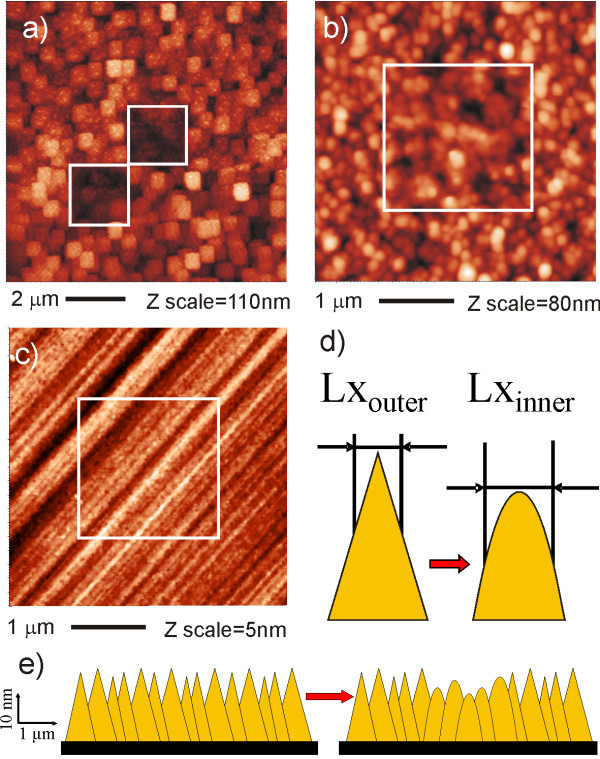
**Wear of diamond substrates in contact mode AFM**. (a) Two 2 × 2 μm^2 ^CM scans with *F *= 3 μN were made in the central area using NSG01 tip with *k *= 3 N/m. Overall image in TM reveal holes with depth *h *= 20 ± 15 nm. (b) CM nanoshaving (2 × 2 μm^2^) image of NCD was made in air using NSG01 tip with applied force *F *= 1.5 μN. Overall image in TM reveal hole with depth *h *= 6 ± 12 nm. (c) CM nanoshaving (1 × 1 μm^2^) on MCD surface was made using NSG01 tip with *F *= 3 μN (*k *= 3 N/m). Overall image in TM (2 × 2 μm^2^) reveals absence of any changes on the surface. (d) The model illustrating wear of NCD grains by Si cantilever. CM nanoshaving makes NCD grains more rounded and their Lx values slightly increases. (e) The model illustrates formation of a depression in the NCD surface by the wear of NCD grains during AFM scanning in CM.

Figure 3 shows the effect of AFM scanning on the diamond substrate itself and compares monocrystalline and NCDs in this aspect. Figure 3c shows that even when quite a high force (*F *= 3 μN) is applied to silicon tip (NSG01, NTM-DT) CM scan does not change the surface of the MCD. Figure 3a shows that similar force can modify the surface of the NCD. Overall image in TM reveals depressions *h *= 20 ± 15 nm deep. During this process the silicon tip is damaged. This is the reason for square like patterns in the image.

Figure 3b shows the results of the CM nanoshaving performed on NCD surface with the new NSG01 tip and applied force of *F *= 1.5 μN. The central area of NCD become lower, Δ*z *= 6 ± 12 nm, RMS roughness in the nanoshaved region decreased by about 1 nm (RMS_outer _= 12.5 ± 0.5 nm, RMS_inner _= 11.5 ± 0.5 nm). Such wear is not observed on MCD using comparable parameters, as shown in Figure 3c. The Lx value of autocorrelation function, which characterizes lateral size of NCD grains, increased after CM scan from Lx_outer _= 110 ± 20 nm to Lx_inner _= 170 ± 20 nm.

**Figure 4:**
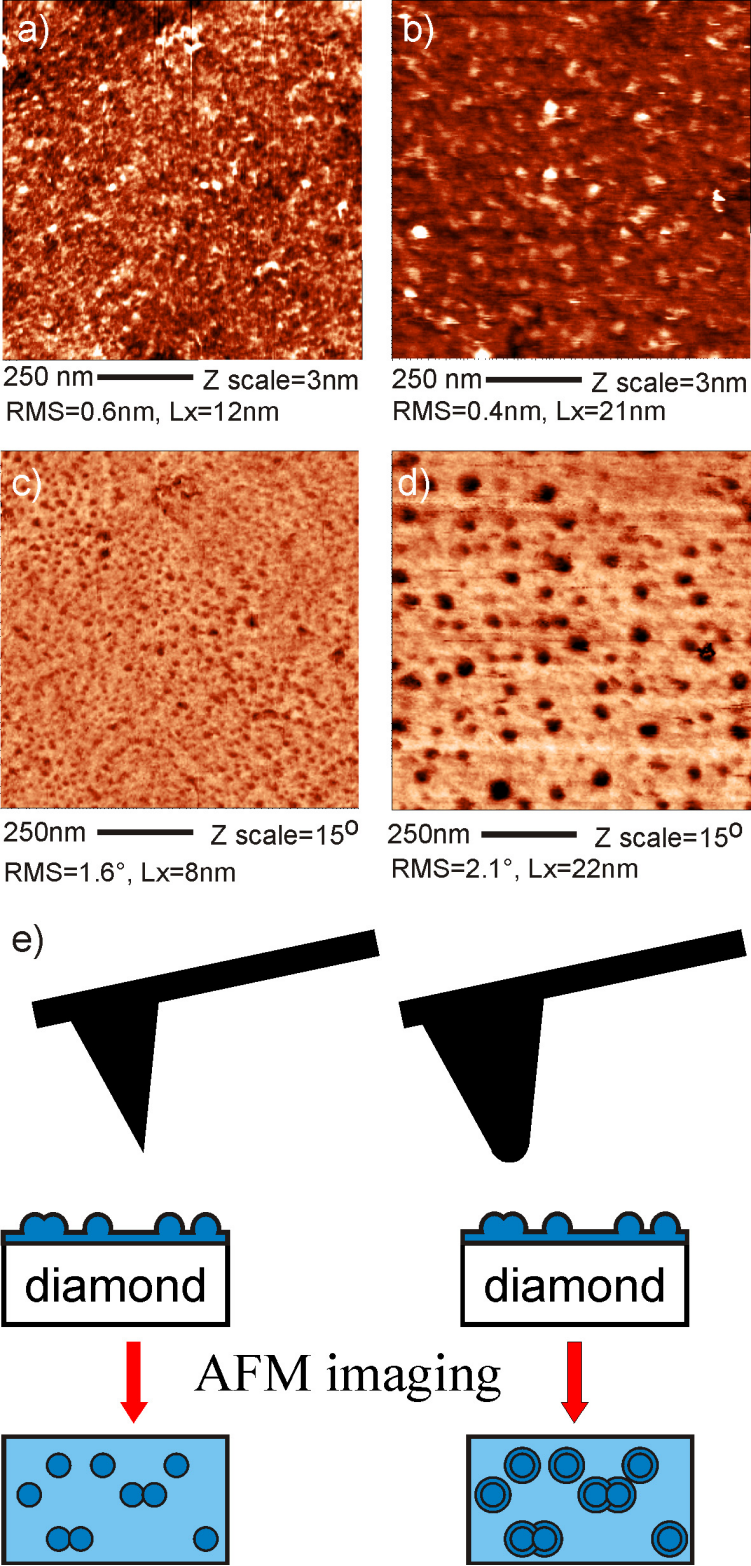
**Influence of the tip radius on AFM topography and phase**. AFM topography (a, b) of FBS layer on H-terminated MCD obtained in solution using cantilevers with different tip radius. Estimated tip radius *r*_a _~ 30 nm, *r*_b _~ 50 nm. Lx_a _= 12 nm, Lx_b _= 21 nm. Corresponding AFM phase images (c, d) show the difference in Lx values for phase (Lx_c _= 8 nm and Lx_d _= 22 nm). (e) The model illustrates the effect of tip shape on the broadening of features in the phase images.

Both morphology and phase images can be influenced also by the tip shape. In Figure 4 one can see the morphology and phase images of FBS layer on H-terminated MCD obtained in TM using the medium *k *cantilevers (*k *= 3 N/m) with a nominal tip radius of *r *~ 10 nm. Dark features (dots) observed in both phase images correspond to protein cores exposed on H-terminated diamond surface [8]. The observed feature size in both topography and phase is different for different cantilevers. The actual tip radius estimated using deconvolution procedure is *r*_a _~ 30 nm for the image (a) and *r*_b _~ 50 nm for the image (b). The sharper tip resolves smaller objects on the surface, Lx_a _< Lx_b _in the topography and Lx_c _< Lx_d _in the phase image. The number of the smaller objects is also higher. RMS values of height and phase signals are more or less the same on both surfaces.

**Figure 5:**
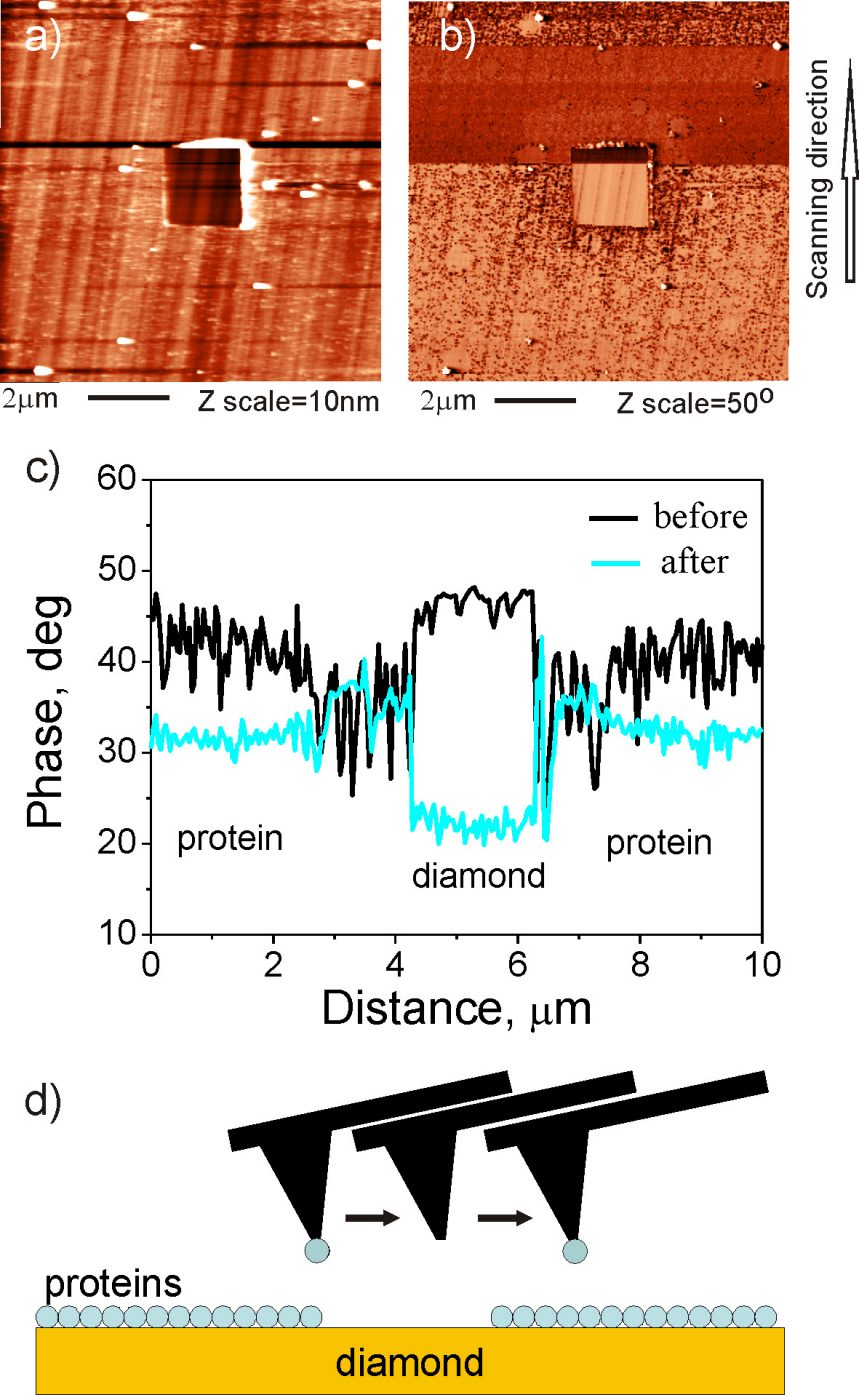
**Influence of tip changes on AFM topography and phase**. AFM image of FBS layer on MCD surface in air observed by Multi75Al (Budget Sensors) cantilever: (a) topography, (b) phase image. (c) The raw phase data showing that contrast between FBS and diamond changed sign during the measurement. (d) The model represents AFM cantilever and protein layer with square opening on diamond surface after the tip has captured a protein from FBS layer. The slow scan direction was from bottom to top of the images.

The tip condition can change during scanning. Figure 5 shows the topography and phase of the FBS layer on MCD, where the central region of 4 × 4 μm^2 ^was nanoshaved using CM and TM. The phase image in Figure 5b and the phase profiles in Figure 5c show that the phase contrast between FBS and diamond changes abruptly both the sign and amplitude during scanning. The topography image looks as if something is dragged on the surface before the phase contrast changed. The image is more "noisy" in that part, but otherwise there are no quantitative changes. After the change of phase the topography image becomes less noisy for some time. Before the scan is finished, it abruptly becomes noisy again. Same effect can be observed in the phase image and profile. Hence the tip has obviously reversibly changed, most likely by releasing and re-capturing some proteins. This process is schematically shown in Figure 5d.

**Figure 6:**
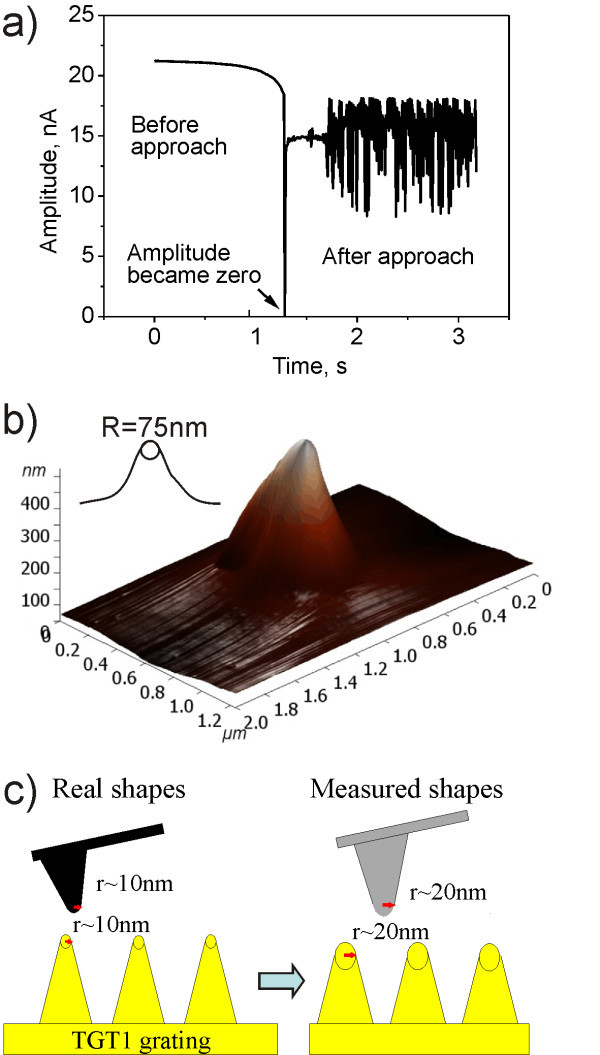
**Tip shape measured after approach to the surface**. (a) Approach curve in TM at Ntegra AFM (NT-MDT) in solution shows that during the first contact amplitude of oscillation *A *became zero. After few seconds the piezotube oscillations cause generations of the amplitude. (b) The Multi75Al tip shape, measured using TGT1 grating. (c) The model illustrates that the measured tip shape is convolution of a real tip shape and grating profile.

The tip condition can be changed already during the initial approach to the surface. Such effect is demonstrated by Figure 6. Figure 6a shows that during cantilever approach in TM the amplitude of its oscillations approached zero. This effect is not affected by cantilever or sample. In addition, there are amplitude oscillations after the approach. Possible reasons of the oscillations are closed-loop system of the scanner and high mass of fluid cell loaded on the scanner. Thus even standard scanner feedback gain can cause oscillations. During these oscillations the amplitude also approaches zero. Several out-of-the-box tips with nominal tip radius of 10 nm were approached to the surface of the TGT1 grating and their shapes were measured by AFM, as shown in Figure 6b. The measured radius *r *was ranging from 50 to 200 nm. The sharpest cantilevers had *r *~ 25 nm. This number actually corresponds well to the nominal tip radius, because the measured tip radius is inherently convolution of the tip and grating profiles, as illustrated by Figure 6c.

## Discussion

We have shown that AFM cantilever spring constant *k *plays crucial role in obtaining reliable results. This is because in TM the spring constant *k *and cantilever oscillation amplitude *A *define the energy of the oscillations. Dissipation of energy Δ*E *that the tip injects into the sample in TM is proportional to *k: *Δ*E *~ *kA*^*2 *^[29]. This relation is similar to the force *F *applied on the sample during CM, which is also proportional to *k*: *F *= *kd*, where *d *is cantilever deflection in CM.

As shown in the Figure 1 the soft cantilevers (*k *= 0.06 N/m) in TM can lead to wrong detection of height in solution and thus should not be used to determine the layer thickness under such conditions. Measurements in CM will remove protein from the surface and therefore should not be used to determine the layer thickness too. Stiff cantilevers (*k *= 120 N/m) are not optimal choice for study of soft biological matter on hard surfaces either because they are too invasive. Based on our results the medium *k *cantilevers are the best choice for *in situ *study of soft matter on hard substrates. TM nanoshaving with those cantilevers is lower than 0.2 nm/scan and there is no problem with the layer height determination.

In this case the amplitude of oscillations of *A*_0 _= 50 nm, which is in the commonly applied range of 10-100 nm, was used to obtain good images. However, if the amplitude is too large (*A*_0 _> 100 nm) the surface structure (height) of the protein layer can be modified even with medium *k *cantilevers as shown in Figure 2. This is because the energy of oscillation and dissipation energy increases with increasing amplitude. On the other hand, this effect can be used to study mechanical and adhesion properties of the protein layers on diamond with different surface terminations [9].

When employed in CM, the medium *k *cantilevers made of silicon can even modify the morphology of NCD films. The data and model in Figure 3 show that after CM the NCD grains became more rounded, Lx value increased, and surface recessed. It is not due to the tip shape as the grains outside the central area remain sharp. It is also not due to deposition of material from the tip because in that case the height of the central area would not become lower. Hence it must be related with mechanical wear of the NCD surface. The decreased RMS by 1 nm corresponds to abrasion of about 3 nm of diamond from tops of the grains.

In addition, surface roughness of NCD films is typically much higher (RMS > 10 nm) compared to MCD (RMS < 1 nm). Such wear and roughness make difficult to use NCD films for characterization of only several nm thin molecular layers. Thus, due to its negligible wear and sub-nanometer roughness, MCD provides a better defined background as a substrate for fundamental studies of diamond-organic interfaces in AFM, and perhaps molecular studies in general.

Condition of the AFM tip is another crucial factor which is responsible for both qualitative and quantitative data quality. Cantilever producers commonly specify tip radius *r *< 10 nm. However, in reality, not all cantilevers provide AFM resolution corresponding to such radius. There are many possible explanations: some cantilevers may be out of the specifications during production, others can be damaged by handling (e.g., electrostatic discharge), AFM approach or measurements.

Features of different dimensions in both topography and phase images on the same FBS layer (see Figure 4) can be explained by different tip radius as shown in the schematic model in Figure 4e. If the tip radius is smaller, than distance between nearest objects, each object will be visible as separate object and its size will depend on the tip radius. If the tip radius is bigger than the distance between features, the features are sensed as one bigger object. On the other hand, some small isolated features may disappear due to averaging effect. Hence it is recommendable to use tips with radius lower than average feature size, check their shape by deconvolution, and test several tips prior to interpretation of the data. It should be noted that some feature broadening is always present in AFM due to non-zero tip radius even for the best tips.

Furthermore, AFM tip can be changed due to collision on the surface during scanning as shown in Figure 5. The collision can result in a release of some material from the tip (be it contamination or material of the tip itself) or in a capture of some material from the surface. That leads to two basic effects. First, it leads to a change of the tip mass and hence resonance frequency *f *and free amplitude *A*_0_. For instance, in the case of material capture the mass is increased, resonance frequency (*f *~ (*k/m*)^1/2^) and free amplitude (*A*_0 _~ 1/*m*) are decreased. Second, it leads to different tip surface interaction and energy dissipation. This effectively changes phase signal. The phase signal corresponds to energy dissipation [29]. The changes of oscillator properties and surface chemistry (protein on the tip surface) can cause changes in interaction between tip and surface (repulsive and attractive regimes) and hence different energy dissipation and change of the phase contrast. Therefore, change of the tip by releasing and capturing proteins from the surface changes the phase contrast as shown in Figure 5. Different quality of the tip-surface interaction can change also AFM topography. Nevertheless the effect is not as pronounced as in the case of AFM phase. It is well known that the AFM phase contrast depends on such scanning parameters as cantilever amplitude and TM set-point [32]. Such changes in phase contrast are controllable and reversible. However, the changes in phase contrast in Figure 5 were due to collisions of the tip with the surface. As such they were uncontrollable and unpredictable. Those changes could lead to incorrect interpretations and should be considered as possible artifact of TM AFM.

AFM tip can also be damaged already during initial approach to the surface and that even in TM where one expects much weaker tip-surface interaction. Unfortunately, approach routine and parameters differ from producer to producer and are often not fully disclosed. In our case, the AFM feedback loop is on and a step motor moves the piezotube with high speed (50 μm/s) until *r*_SP _~ 0.8. Then the motor moves the piezotube with slow speed (0.6 μm/s) to the value given by the amplitude set-point. This method is relatively fast. Its drawback is possibility of crashing the tip if the last step made by the step motor is too fast or too large and the scanner feedback can not compensate this movement. However, too high feedback gain can cause oscillations in the amplitude as evidenced in Figure 6a. These oscillations can also damage the tip.

The only way how to systematically monitor the actual approach quality is to monitor the cantilever amplitude during approach. If the amplitude drops close to zero, albeit briefly, the tip may be damaged as evidenced in Figure 6. This is in particular critical for silicon-based tips on hard substrates such as diamond. Moreover, if the feedback value is too high during approach, tip will start to oscillate immediately after contact. These oscillations may also damage the tip. If the FB value during approach is too low, the scanner response will be slow and cantilever may hit the surface as well.

To estimate force which is applied to the tip during approach one can assume that tip is pressed against the surface for distance equal to free oscillation amplitude. It corresponds to the force which is applied to cantilever during CM measurements: *F *= *kA*_0_. For example *F *~ 600 nN for NSG01 cantilever at *A*_0 _= 60 nm in the case of Figure 6. So strong interaction can obviously damage the very tip.

Thus, for studies where dimensions and/or phase contrast on sub-100 nm scale are important, a calibration is recommendable. This can be accomplished during measurements by using predefined objects or regions on the surface with known dimensions and/or phase contrast. Another possibility is to use the same tip for all measurements and monitor whether the tip has changed or not. Silicon-based tips may get blunt on hard or rough substrates already after few scans, so it may be better to use diamond-coated or bulk diamond tips which are more durable [33] but at the cost of typically lower resolution. The above guidelines actually apply and may be helpful in general for all AFM regimes, both basic and advanced such as current-sensing AFM [34].

## Conclusions

AFM in TM and CM regimes were employed for high resolution studies of FBS proteins on diamond in solution and in air. Various effects in morphology and phase measurements related to the cantilever spring constant, amplitude of tip oscillations, surface approach, and tip shape were demonstrated and discussed based on the proposed models. We also suggested methods how to choose suitable cantilever, perform fine tuning of scanning parameters in TM, recognize and minimize various artifacts, and obtain reliable AFM images. AFM cantilevers with medium *k *~ 3 N/m and amplitudes around *A*_0 _~ 50 nm were identified as the most suitable for protein-diamond characterizations, in particular in solution. As a substrate, MCD provided well-defined background for AFM studies due to its negligible wear and sub-nanometer surface roughness compared to, e.g., NCD. Due to the possibility of tailoring diamond surface properties by various atomic terminations, it may have even broader application for fundamental studies of molecules on surfaces in general. Based on the results of this study, researchers in fields of life sciences, bio-physics, and bio-technologies may better optimize and understand AFM measurements and avoid incorrect conclusions.

## Abbreviations

AFM: atomic force microscopy; CM: contact mode; FBS: fetal bovine serum; MCD: monocrystalline diamond; NCD: nanocrystalline diamond; RMS: root mean square; SAOS-2: sarcoma osteogenic; TM: tapping mode.

## Competing interests

The authors declare that they have no competing interests.

## Authors' contributions

BR coordinated the study, wrote the manuscript and participated in discussion and interpretation of data. EU performed sample preparation, AFM measurements, participated in discussion and interpretation of data and drafted the manuscript. AK performed H-termination of the diamond surfaces and participated in discussions related with diamond surfaces. All authors read and approved the final manuscript.

## Authors' information

BR born in 1973 in Prague, Czech Republic, graduated from Physics at the Faculty of Mathematics and Physics at the Charles University in Prague, continued with PhD at Academy of Sciences of the Czech Republic (ASCR) in a group of Dr. Jan Kočka on the charge transport in amorphous and microcrystalline silicon with high lateral resolution by the use of scanning probe techniques. During his PhD he spent several research stays in a group of Prof. Martin Stutzmann at the Walter Schottky Institut, Technische Universität München. There he worked with Dr. Christoph Nebel on development of large grain silicon thin films using interference laser crystallization of amorphous silicon layers and on their investigation by laser beam induced currents with a sub-micrometer lateral resolution, with a special view to optical and electronic properties of grain boundaries. After receiving PhD degree in 2001, he continued in the group of Prof. Stutzmann as a postdoctoral researcher on the project for diamond devices and sensors where he focused on a study and modification of hydrogen terminated diamond surfaces and their electrolytic interfaces. In 2002 he joined the Nanotechnology Group at the Swiss Federal Institute of Technology, where he was working on a guided assembly of colloidal nanoparticles at solid state surfaces. Since 2004, he worked at the Diamond Research Center of AIST in Tsukuba, Japan, doing research on surface (bio)-functionalized diamond devices. In 2006 he became research team leader and Purkyně Fellow at the Institute of Physics ASCR in Prague, Czech Republic. His research team is focused on nano-interfaces of semiconductors and organic materials towards opto-electronic and bio-electronic applications. Main interests lie in characterization and modification of material, electronic, and chemical properties by local probe techniques as well as in guided assembly of organic and inorganic nanostructures. He is the author or co-author of over 100 scientific articles in international peer-reviewed journals that were cited more than 600 times and of several patents (3) and patent applications.

EU born in 1981 in Ekaterinburg, Russia, graduated from Physics at the Moscow State University in Moscow, Russia, continued with PhD at the Moscow State University in a group of Dr. Igor Yaminsky on the AFM study of aggregation of polymers on different substrates. During his PhD he spent 1 year in a group of Prof. Yuri Lyubchenko at the University Nebraska Medical Center, Omaha, USA. There he worked with Prof. Yuri Lyubchenko on investigation of amyloid beta peptide conformation using force spectroscopy. After receiving PhD degree in 2007, he continued in the group of Dr. Bohuslav Rezek on the project for functional hybrid nanosystems of semiconductors and metals with organic materials. He is the author or co-author of over 10 scientific articles in international peer-reviewed journals.

AK born in 1971 Nitra, Slovak Republic, graduated from Microelectronics at the Department of Microelectronics at the Slovak Technical University, Bratislava (1995). In 2001, he received his PhD degree in Electronics with main focus on the growth of diamond thin films by a hybrid hot-filament chemical vapor deposition (CVD) technique in the group of Dr. S. Bederka. He acquired experience in semiconductor processing (optical lithography, plasma etching, deposition of metal contacts, etc.) during his postdoctoral position at University of Ulm (Germany). During his short stay at the Institute of Physics ASCR in 2001, he start up the microwave plasma CVD system for diamond thin film growth at high pressures (up to 250 mbars). In 2002 he joined a.o.t. GmbH Company (Austria) where he focused on large area deposition of diamond films by modified hot-filament CVD process. He actively participated in establishing R&D projects within the national and international collaboration. In 2005 he joined Institute of Physics ASCR (Czechia) with the main focus on nucleation/seeding techniques and low temperature growth of diamond films on variety substrates (Si, glass, Al, SiC, etc.). In 2008 he visited group of Prof. Haenen at IMO IMEC (Hasselt, Belgium), where he gained experience in the growth of boron-doped diamond films. Since 2009, he is the group leader of Diamond and Carbon nano-Structures Laboratory and Purkyně Fellow at the Institute of Physics ASCR (Czechia). His research activity is focused on growth of diamond thin films and microstructures across large area, diamond nanostructuring by dry plasma processes, low temperature plasma processes, and development of novel diamond-based electronic and optical devices. He is an author or co-author of over 78 scientific articles in internationally peer-reviewed journals that were cited more than 280 times, 2 patents and several patent applications.
